# PCSK9 inhibitors improve lipid profile and hepatic steatosis surrogate indicators in patients with MAFLD and type 2 diabetes

**DOI:** 10.3389/fmed.2026.1756998

**Published:** 2026-03-05

**Authors:** Qingna Zhou, Xiaoxia Liu, Yunzhao Tang, Congqing Pan, Xuena Bi

**Affiliations:** 1 Department of Cardiovascular Medicine, NHC Key Lab of Hormones and Development and Tianjin Key Lab of Metabolic Diseases, Tianjin Medical University Chu Hsien-I Memorial Hospital & Institute of Endocrinology, Tianjin, China; 2 Department of Ultrasound, NHC Key Lab of Hormones and Development and Tianjin Key Lab of Metabolic Diseases, Tianjin Medical University Chu Hsien-I Memorial Hospital & Institute of Endocrinology, Tianjin, China; 3 Department of Integrated Traditional Chinese and Western Medicine, NHC Key Lab of Hormones and Development and Tianjin Key Lab of Metabolic Diseases, Tianjin Medical University Chu Hsien-I Memorial Hospital & Institute of Endocrinology, Tianjin, China; 4 Department of Cardiovascular Medicine, Beichen Hospital of Nankai University, Tianjin, China

**Keywords:** metabolic dysfunction-associated fatty liver disease (MAFLD), non-alcoholic fatty liver disease (NAFLD), proprotein convertase subtilisin kexin type-9 inhibitor (PCSK9i), sodium-glucose co-transporter 2 inhibitor (SGLT-2i), type 2 diabetes mellitus (T2DM)

## Abstract

**Objective:**

To investigate the impact of proprotein convertase subtilisin kexin type-9 inhibitor (PCSK9i) on patients with metabolic dysfunction-associated fatty liver disease (MAFLD) combined with type 2 diabetes mellitus (T2DM).

**Methods:**

This retrospective study reviewed the clinical data of 60 inpatients with MAFLD combined with T2DM from the electronic medical record (EMR) system. According to the medical records, all patients were categorized into the Control group (*n* = 30, atorvastatin 20 mg QN) and the PCSK9i group (*n* = 30, evolocumab injection 140 mg Q2W in addition to atorvastatin). Body mass index (BMI), glycemic control, hepatic fibrosis and steatosis surrogate indicators such as aspartate aminotransferase to platelet ratio index (APRI), fibrosis-4 index (FIB-4), fatty liver index (FLI) and controlled attenuation parameter (CAP), and lipid profiles, including total cholesterol (TC), triglycerides (TG), high-density lipoprotein cholesterol (HDL-C), low-density lipoprotein cholesterol (LDL-C), were analyzed at baseline and the 12-week follow-up in both groups. Multivariable regression analyses for changes in hepatic fibrosis and steatosis surrogate indicators were performed.

**Results:**

At the 12-week follow-up, both groups exhibited significant reductions in lipid levels, with the PCSK9i group demonstrating greater decreases in TC (48.65 vs. 23.32%) and LDL-C (25.84 vs. 21.09%) compared to the Control group (*P* < 0.05). Meanwhile, the PCSK9i group exhibited significantly greater reductions in CAP (22.41 vs. 15.60%) and FLI (27.72 vs. 13.77%) in unadjusted analyses (both *P* < 0.05). Multivariable regression analyses demonstrated the superior improvement in CAP and FLI observed with PCSK9-i is independent of concomitant sodium-glucose co-transporter 2 inhibitor (SGLT-2i) therapy.

**Conclusion:**

PCSK9i effectively reduced hepatic steatosis surrogate scores (FLI, CAP) and lipid levels (TC, LDL-C) in patients with MAFLD combined with T2DM.

## 1 Introduction

Fatty liver disease (FLD), a leading global health concern, is traditionally classified into non-alcoholic fatty liver disease (NAFLD) and alcoholic fatty liver disease (AFLD). In 2020, an international consensus redefined NAFLD as metabolic dysfunction-associated fatty liver disease (MAFLD) to better reflect its association with metabolic abnormalities (1). The rising prevalence and incidence of MAFLD have made it the primary cause of chronic liver disease worldwide (2). MAFLD is recognized as the hepatic manifestation of metabolic syndrome, which requires the exclusion of other liver diseases, with more than 5% liver fat accumulation and no excessive alcohol consumption (defined as 20 g/day for women and 30 g/day for men) (3). Additionally, the diagnosis of MAFLD requires the presence of at least one of the following criteria: overweight/obesity, type 2 diabetes, or evidence of metabolic dysfunction. Secondary causes of fat accumulation, such as alcoholic, viral hepatitis, or autoimmune hepatitis, must also be excluded. MAFLD mostly coexists with health problems such as obesity, T2DM and insulin resistance, resulting into serious adverse effects on liver and extrahepatic clinical outcomes (4). Notably, the prevalence of MAFLD in patients with T2DM is as high as 70%, and MAFLD patients exhibit a significantly increased risk of developing T2DM (5). Despite these associations, no specific pharmacological therapies for MAFLD are currently available. The pathogenesis of MAFLD is multifaceted, involving insulin resistance, lipotoxicity, inflammation, cytokine imbalance, innate immune activation, and alterations in gut microbiota (6). While combination therapies targeting glucose and lipid metabolism, hepatic inflammation, and fibrosis show promise, the management of MAFLD in patients with T2DM remains underaddressed in clinical practice. This gap underscores the urgent need to identify novel molecular targets and therapeutic strategies. For patients with MAFLD combined with T2DM, the risk of cardiovascular disease, malignant tumors, and all-cause mortality is significantly elevated, further highlighting the clinical significance of addressing this comorbidity.

As a key regulator of LDL metabolism, PCSK9 binds to the LDL receptor (LDL-R) on hepatocytes, leading to its lysosomal degradation and subsequently increasing plasma LDL-C levels. Emerging evidence implicates PCSK9 in the pathogenesis of MAFLD through dual mechanisms: promoting lipid accumulation and modulating hepatic inflammatory responses. Recent studies have indicated that PCSK9 plays a role in the pathogenesis of MAFLD, with high levels of PCSK9 in the liver or circulation increasing lipid storage in muscles and the liver, fat energy storage, and the storage and secretion of fatty acids and TG in the liver, thereby promoting the development of MAFLD (7, 8). Specifically, elevated hepatic or circulating PCSK9 levels have been shown to enhance intracellular lipid storage via upregulation of fatty acid synthase (FASN) and acetyl-CoA carboxylase (ACC), while simultaneously impairing mitochondrial β-oxidation through AMPK/mTOR signaling dysregulation (9). Paradoxically, PCSK9 also exhibits a protective role by mediating CD36 receptor degradation, thereby limiting hepatic fatty acid uptake and triglyceride (TG) accumulation—a mechanism potentially relevant to attenuating steatosis progression (10, 11).

Clinical and preclinical studies present conflicting findings regarding PCSK9 inhibition in MAFLD. PCSK9i is monoclonal antibody, such as alirocumab or evolocumab, that bind to plasma PCSK9, preventing LDL-R degradation and thereby reducing plasma LDL-C levels (12). Clinically, PCSK9i is used for lipid management in patients with familial hypercholesterolemia and coronary artery disease (ASCVD), significantly reducing cholesterol levels and cardiovascular risk (13, 14). Despite lowering plasma LDL-C levels, PCSK9i may promote higher lipid accumulation and the progression of MAFLD (15). PCSK9-deficient mice after feeding a high-fat diet may develop severe liver steatosis and fibrosis, atherosclerosis (16, 17). Interestingly, some studies have reached different conclusions, suggesting that PCSK9i may reduce liver fat accumulation, inflammation, and fibrosis (7), slow or even completely alleviate MAFLD (18).

Despite these insights, the translational potential of PCSK9i in MAFLD comorbid with T2DM remains underexplored. Considering current practice prioritize PCSK9i use in cardiovascular risk reduction, yet their metabolic benefits warrant further elucidation. This study seeks to bridge this knowledge gap by evaluating the effects of PCSK9i on lipid profiles, hepatic steatosis, and metabolic parameters in patients with MAFLD and T2DM, thereby providing mechanistic and clinical evidence for their therapeutic utility in this high-risk population.

## 2 Methods

### 2.1 Study design and population

**Figure 1:**
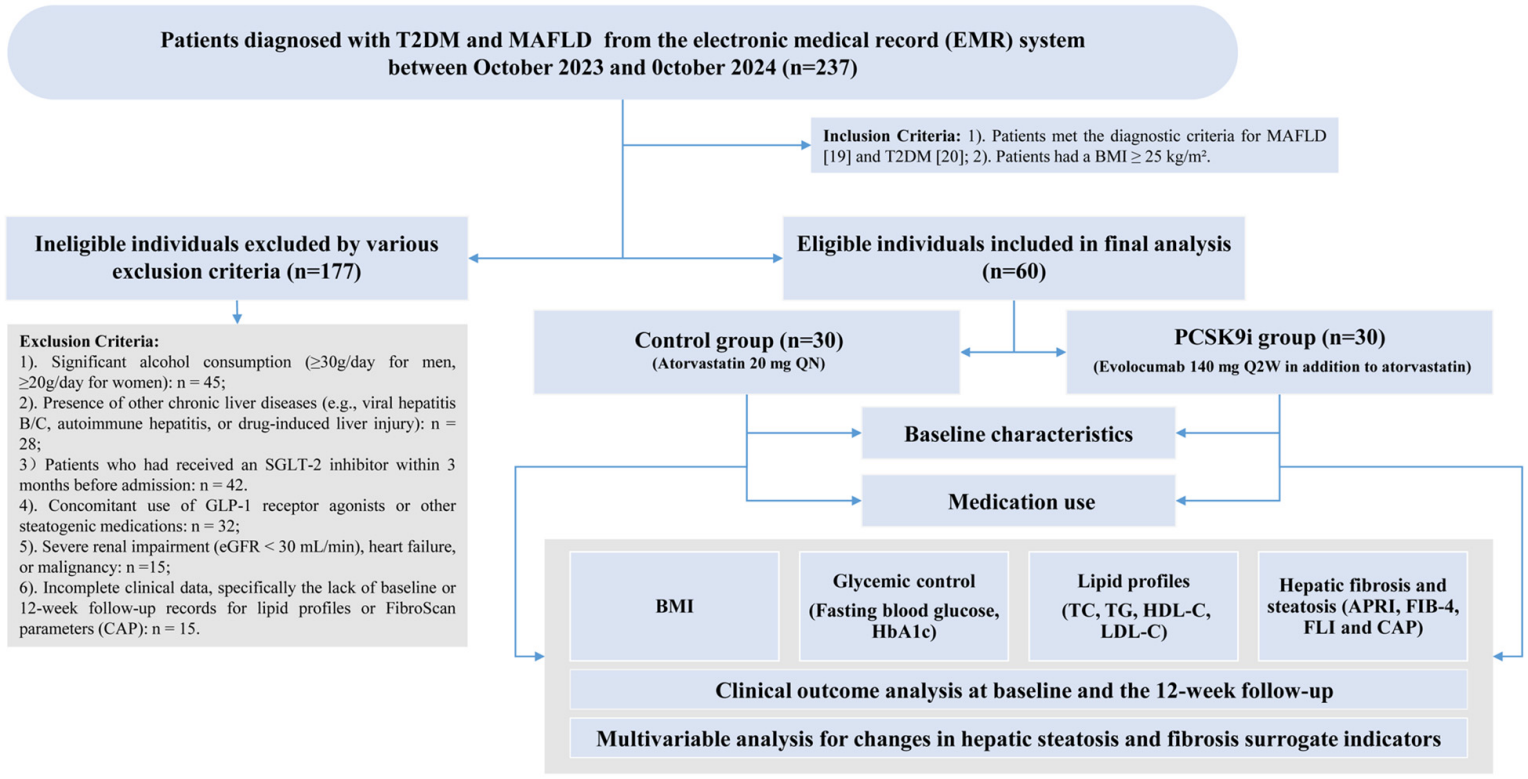
Study workflow diagram..

This was a single-center, retrospective cohort study. Medical records of all patients discharged from Tianjin Medical University Chu Hsien-I Memorial Hospital between October 2023 and October 2024 were retrospectively reviewed. The detailed screening process was illustrated in Figure 1. Finally, 60 eligible inuals were identified from 237 patients diagnosed with both T2DM and MAFLD. No prospective enrolment or additional study visits were performed. The study were approved by the ethics committee of our hospital (NO. ZXYJNYYkMEC2025-04) on March 27, 2025 and conformed to the 1964 Helsinki Declaration and its later amendments or comparable ethical standards. A written informed consent was waived from each participant due to the nature of retrospective study.

According to the documented medical regimens, patients were allocated to the “PCSK9-inhibitor (PCSK9i) group” if the discharge medication list contained evolocumab injection (140 mg every 2 weeks) in addition to atorvastatin 20 mg nightly; they were allocated to the “Control group” if the list contained atorvastatin 20 mg nightly alone. Treatment decisions had been made by the attending physicians according to routine clinical practice and were extracted verbatim from the electronic medical record (EMR). Baseline data were defined as the last record prior to discharge; 12-week Follow-up was defined as the first outpatient encounter. No extra investigations were requested for the purpose of this analyses.

### 2.2 Inclusion and exclusion criteria

Inclusion Criteria: (1). Patients met the diagnostic criteria for MAFLD (19) and T2DM (20); (2). Patients had a BMI ≥ 25 kg/m^2^. (3) Patients received atorvastatin alone or in combination with evolocumab.

Exclusion Criteria: (1). Significant alcohol consumption (≥30 g/day for men, ≥20 g/day for women); (2). Presence of other chronic liver diseases (e.g., viral hepatitis B/C, autoimmune hepatitis, or drug-induced liver injury); (3). Patients who had received an SGLT-2 inhibitor within 3 months before admission; (4). Concomitant use of GLP-1 receptor agonists or other steatogenic medications; (5) Severe renal impairment (eGFR < 30 ml/min), heart failure, or malignancy; (6). Incomplete clinical data, specifically the lack of baseline or 12-week follow-up records for lipid profiles or FibroScan parameters (CAP).

### 2.3 Clinical data collection

Baseline characteristics including age, gender, body mass index (BMI), smoking history, duration of diabetes, history of hypertension, history of hyperuricemia, history of coronary heart disease were collected. Moreover, blood glucose levels [hemoglobin A1c (HbA1c) and fasting blood glucose (FBG)], renal function (creatinine), liver function [alanine aminotransferase (ALT), aspartate aminotransferase (AST), platelet (PLT), lipid profiles total cholesterol (TC), triglycerides (TG), high-density lipoprotein cholesterol (HDL-C), low-density lipoprotein cholesterol (LDL-C)], aspartate aminotransferase to platelet ratio index (APRI), fibrosis-4 index (FIB-4) was measured by FibroScan transient elastography, as well. Fatty liver index (FLI) was calculated using the following formula. During the follow-up, the medication use were also recorded.


$$\begin{array}{l} FLI=\\ \;\left[e^{0.953\times ln\left(TG\right)+0.139\times BMI+0.718\times ln\left(r-GGT\right)+0.053\times WC-15.745}\right]/\\ \;\left[1+e^{0.953\times ln\left(TG\right)+0.139\times BMI+0.718\times ln\left(r-GGT\right)+0.053\times WC-15.745}\right]\\ \;\times 100 \end{array}$$

### 2.4 Clinical outcome measures

The clinical outcomes were to assess the weight changes of BMI, glycemic control (HbA1c), lipid profiles (TC, TG, HDL-C, LDL-C), and hepatic fibrosis and steatosis surrogate indicators (APRI, FIB-4, FLI and CAP) at baseline and the 12-week follow-up.

### 2.5 Statistical analyses

All analyses were performed in R (version 4.3.2; R Foundation for Statistical Computing, Vienna, Austria). After cohort identification, a *post-hoc* power calculation (pwr package) confirmed that the observed LDL-C effect (Cohen's *d* = 0.94, *n* = 30 per group) provided 96.4% power at α = 0.05 (two-tailed). Normally distributed continuous variables are expressed as mean ± standard deviation (SD) and compared between groups with independent-samples *t-*tests; categorical variables are summarized as *n* (%) and analyzed using Chi-square tests or Fisher's exact tests. Multivariate analyses was conducted to attenuate residual confounding. Two-sided *P* < 0.05 was considered statistically significant.

## 3 Results

### 3.1 Comparison of baseline characteristics

As shown in Table 1, baseline characteristics between the Control group (*n* = 30) and the PCSK9i group (*n* = 30) were compared. The baseline creatinine levels were significantly higher in the PCSK9i group than the Control group (73.72 ± 12.93 vs. 64.11 ± 15.58 μmol/L, *P* = 0.012). There was no significant difference in other baseline characteristics (*P* > 0.05).

**Table 1:** Baseline characteristics between the control group and the PCSK9 inhibitor group.

**Index**	**Control group (*n* = 30)**	**PCSK9i group (*n* = 30)**	**Mean difference (95% CI)**	**χ^2^/*t***	***P-*value**
Age (years)	61.03 ± 11.97	64.67 ± 9.76	−3.64 (−9.29, 2.01)	−1.291	0.202
Female (*n*, %)	23 (76.7%)	17 (56.7%)	–	2.7	0.1
BMI (kg/m^2^)	30.87 ± 6.07	31.03 ± 5.86	−0.16 (−3.24, 2.92)	−0.104	0.918
Smoking history (*n*, %)	11 (36.7%)	16 (53.3%)	–	1.684	0.194
Duration of diabetes	10.15 ± 6.60	8.62 ± 6.09	1.53 (−1.75, 4.81)	0.933	0.355
Hypertension (*n*, %)	15 (50.0%)	17 (56.7%)	–	0.268	0.605
Hyperuricemia (*n*, %)	5 (16.7%)	4 (13.3%)	–	0.131	0.718
Coronary heart disease	13 (43.3%)	15 (50.0%)	–	0.268	0.605
HbA1c (%)	8.03 ± 1.27	8.47 ± 1.34	−0.44 (−1.12, 0.24)	−1.305	0.197
FBG (mmol/L)	8.01 ± 1.46	8.53 ± 2.05	−0.52 (−1.44, 0.40)	−1.133	0.262
Creatinine (μmol/L)	64.11 ± 15.58	73.72 ± 12.93	−9.61 (−17.02, −2.20)	−2.599	0.012^*^
ALT (U/L)	20.74 ± 18.36	30.40 ± 22.33	−9.66 (−19.95, 0.63)	−1.86	0.068
AST (U/L)	22.40 ± 16.71	31.00 ± 20.45	−8.60 (−18.01, 0.81)	−1.81	0.076
PLT ( × 10^9^/L)	147.18 ± 23.20	142.94 ± 15.48	4.24 (−5.88, 14.36)	0.83	0.41
TG (mmol/L)	2.46 ± 1.64	2.94 ± 1.67	−0.48 (−1.34, 0.38)	−1.123	0.266
TC (mmol/L)	6.56 ± 2.62	7.46 ± 1.64	−0.90 (−1.99, 0.19)	−1.595	0.116
LDL-C (mmol/L)	3.26 ± 1.23	3.84 ± 1.45	−0.58 (−1.27, 0.11)	−1.67	0.1
HDL-C (mmol/L)	1.04 ± 0.24	1.09 ± 0.31	−0.05 (−0.19, 0.09)	−0.7	0.487
APRI	0.157 ± 0.075	0.164 ± 0.102	−0.007 (−0.05, 0.04)	−0.303	0.763
FIB-4	2.41 ± 1.43	2.54 ± 2.06	−0.13 (−1.05, 0.79)	−0.284	0.777
CAP (dB/m)	276.4 ± 63.5	285.1 ± 62.7	−8.70 (−41.3, 23.9)	−0.534	0.595
FLI	42.12 ± 6.98	43.21 ± 8.67	−1.09 (−5.16, 2.98)	−0.536	0.594

PCSK9i, proprotein convertase subtilisin kexin type-9 inhibitor; BMI, body mass index; HbA1c, hemoglobin A1c; ALT, alanine aminotransferase; AST, aspartate aminotransferase; PLT, platelet; TG, triglycerides; TC, total cholesterol; LDL-C, low-density lipoprotein cholesterol; HDL-C, high-density lipoprotein cholesterol; CAP, controlled attenuation parameter; FLI, fatty liver index; APRI, aspartate aminotransferase to platelet ratio index; FIB-4, fibrosis-4 index. ^*^P < 0.05 indicates statistical significance.

### 3.2 Comparison of medication use during 12-week follow-up

The medication use between the Control group and the PCSK9i group was also analyzed during 12-week follow-up (Table 2). There was no significantly differences in other medication use (*P* > 0.05), except PCSK9i.

**Table 2:** Medication use between the control group and the PCSK9 inhibitor group.

**Medication use**	**Control group (*n* = 30)**	**PCSK9i group (*n* = 30)**	**Mean difference (95% CI)**	**χ^2^**	***P*-value**
Atorvastatin (*n*, %)	30 (100.0%)	30 (100.0%)	−11.5%, 11.5%	–	1.000
PCSK9i (*n*, %)	0 (0.0)	30 (100.0%)	−1.00%, −0.88%	–	0.000
Fenofibrate (*n*, %)	5 (16.7%)	4 (13.3%)	−15.9%, 22.8%	0.131	0.718
Ezetimibe (*n*, %)	8 (26.7%)	8 (26.7%)	−22.5%, 22.5%	–	1.000
SGLT2i (*n*, %)	18 (60.0%)	22 (73.3%)	−36.4%, 10.3%	1.200	0.273
Insulin preparations (*n*, %)	15 (50.0%)	13 (43.3%)	−18.3%, 30.9%	0.268	0.605
Metformin (*n*, %)	26 (86.7%)	26 (86.7%)	−18.8%, 18.8%	–	1.000
Miglitol (*n*, %)	20 (66.7%)	22 (73.3%)	−29.7%, 16.3%	0.314	0.573
Aspirin (*n*, %)	13 (43.3%)	15 (50.0%)	−30.9%, 18.3%	0.268	0.605
Febuxostat (*n*, %)	5 (16.7%)	4 (13.3%)	−15.9%, 22.8%	0.131	0.718

PCSK9i, proprotein convertase subtilisin kexin type-9 inhibitor; SGLT-2i, sodium-glucose co-transporter 2 inhibitor.

### 3.3 Clinical outcome analyses

The clinical outcomes were further analyzed (Table 3). Both groups showed improvements in glycemic control and BMI, and there were no significant differences in the reduction of BMI (*P* = 0.067) or FBG (*P* = 0.308) between the two groups. However, the PCSK9i group exhibited a significantly greater reduction in HbA1c compared to controls (2.20 vs. 1.62%, *P* = 0.029). Importantly, the specific impact of PCSK9i was most pronounced in lipid profiles. The reduction in TG was significantly greater in the PCSK9i group (1.39 vs. 0.63 mmol/L, *P* = 0.015), consistent with the robust reductions observed in TC and LDL-C (*P* < 0.001). Furthermore, the PCSK9i group achieved a significantly larger reduction in FLI (11.98 vs. 5.80, *P* < 0.001) and CAP (63.90 vs. 41.90 dB/m, *P* = 0.025) at the 12-week follow-up observation period.

**Table 3:** Summarizes the clinical outcomes and the magnitude of changes from baseline.

**Index**	**Control group (*n* = 30)**	**PCSK9i group (*n* = 30)**	**Mean difference (95% CI)**	***t***	***P-*value**
**Anthropometrics**					
**BMI (kg/m** ^2^ **)**					
12-week follow-up	29.10 ± 4.14	30.35 ± 2.95	1.25 (−0.61, 3.11)	1.346	0.184
^Δ^Reduction	1.77 ± 2.50	0.68 ± 2.00	−1.09 (−2.26, 0.08)	−1.865	0.067
**Glycemic Control**					
**FBG (mmol/L)**					
12-week follow-up	6.96 ± 0.97	7.12 ± 1.18	0.16 (−0.39, 0.71)	0.573	0.569
^Δ^Reduction	1.05 ± 1.20	1.41 ± 1.50	0.36 (−0.34, 1.06)	1.028	0.308
**HbA1c (%)**					
12-week follow-up	6.41 ± 1.35	6.27 ± 1.19	−0.14 (−0.80, 0.52)	−0.426	0.672
^Δ^Reduction	1.62 ± 0.90	2.20 ± 1.10	0.58 (0.06, 1.10)	2.235	0.029^*^
**Lipid profiles**					
**TG (mmol/L)**					
12-week follow-up	1.83 ± 0.68	1.55 ± 0.57	−0.28 (−0.60, 0.04)	−1.725	0.09
^Δ^Reduction	0.63 ± 1.10	1.39 ± 1.20	0.76 (0.16, 1.36)	2.556	0.015^*^
**TC (mmol/L)**					
12-week follow-up	5.03 ± 1.49	3.83 ± 0.68	−1.20 (−1.79, −0.61)	−4.045	< 0.001^*^
^Δ^Reduction	1.53 ± 2.05	3.63 ± 1.88	2.10 (1.09, 3.11)	4.133	< 0.001^*^
**LDL-C (mmol/L)**					
12-week follow-up	3.03 ± 0.76	2.64 ± 0.62	−0.39 (−0.74, −0.03)	−2.176	0.034^*^
^Δ^Reduction	0.23 ± 0.95	1.20 ± 1.10	0.97 (0.44, 1.50)	3.649	< 0.001^*^
**HDL-C (mmol/L)**					
12-week follow-up	0.97 ± 0.87	1.03 ± 0.23	0.06 (−0.27, 0.39)	0.366	0.717
^Δ^Reduction	0.07 ± 0.60	0.06 ± 0.25	−0.01 (−0.25, 0.23)	−0.084	0.933
**Liver stiffness and steatosis**					
**APRI**					
12-week follow-up	0.15 ± 0.06	0.14 ± 0.09	−0.01 (−0.05, 0.03)	−0.505	0.616
^Δ^Reduction	0.01 ± 0.05	0.02 ± 0.08	0.01 (−0.02, 0.04)	0.582	0.563
**FIB-4**					
12-week follow-up	2.21 ± 1.76	2.14 ± 2.98	−0.07 (−1.33, 1.19)	−0.111	0.912
^Δ^Reduction	0.20 ± 1.05	0.40 ± 1.50	0.20 (−0.47, 0.87)	0.598	0.552
**FLI**					
12-week follow-up	36.32 ± 4.54	31.23 ± 5.43	−5.09 (−7.67, −2.51)	−3.938	< 0.001^*^
^Δ^Reduction	5.80 ± 3.50	11.98 ± 6.20	6.18 (3.59, 8.77)	4.767	< 0.001^*^
**CAP (dB/m)**					
12-week follow-up	234.51 ± 45.81	221.20 ± 31.70	−13.31 (−33.74, 7.12)	−1.309	0.196
^Δ^Reduction	41.90 ± 28.12	63.90 ± 18.25	22.00 (7.73, 34.31)	3.597	0.025^*^

PCSK9i, proprotein convertase subtilisin kexin type-9 inhibitor; BMI, body mass index; HbA1c, hemoglobin A1c; TG, triglycerides; TC, total cholesterol; LDL-C, low-density lipoprotein cholesterol; HDL-C, high-density lipoprotein cholesterol; CAP, controlled attenuation parameter; FLI, fatty liver index; APRI, aspartate aminotransferase to platelet ratio index; FIB-4, fibrosis-4 index. ^Δ^ indicates the reduction from baseline to the 12-week follow-up. 12-week follow-up vs. Baseline ^*^P < 0.05 indicates statistical significance.

### 3.4 Multivariable regression analyses

To rigorously exclude the potential confounding effects of concomitant medications, particularly SGLT2i, multivariable linear regression analyses were conducted (Table 4). The changes in hepatic steatosis and fibrosis surrogate indicators during the 12-week observation period (^Δ^CAP, ^Δ^FLI, ^Δ^APRI, and ^Δ^FIB-4) were defined as dependent variables. The groups (PCSK9i vs. Control) was included as the primary independent variable. Covariates entered into the adjustment model included SGLT-2i use, age, sex, BMI, HbA1c, creatinine and the baseline value of each respective outcome. After adjustment, PCSK9i use remained independently associated with greater reductions in hepatic steatosis surrogate markers, specifically ^Δ^CAP (β = −18.6, 95% CI: −32.1 to −5.1, *P* = 0.008) and ^Δ^FLI (β = −6.8, 95% CI: −11.5 to −2.1, *P* = 0.006). In contrast, SGLT2i use was not statistically associated with changes in CAP or FLI (*P* > 0.05). Regarding fibrosis indicators, although PCSK9i use showed a trend toward improvement in ^Δ^APRI (β = −0.021) and ^Δ^FIB-4 (β = −0.19), these associations did not reach statistical significance in the adjusted model (*P* > 0.05). These findings demonstrated that within the 12-week observation period, the independent therapeutic efficacy of PCSK9i was most pronounced in ameliorating hepatic steatosis surrogate markers CAP and FLI.

**Table 4:** Multivariable analyses for changes in hepatic steatosis and fibrosis surrogate indicators.

**Dependent variable**	**Independent variable**	**β (95% CI)**	**Std. β**	***P*-value**
^Δ^CAP (dB/m)	PCSK9i use	−18.6 (−32.1, −5.1)	−0.38	0.008^*^
	SGLT2i use	−4.2 (−16.8, 8.4)	−0.09	0.51
	Baseline CAP	−0.41 (−0.62, −0.20)	−0.44	< 0.001^*^
	Age	0.12 (−0.38, 0.62)	0.04	0.63
	BMI	0.58 (−0.31, 1.47)	0.15	0.19
	HbA1c	0.94 (−1.72, 3.60)	0.1	0.48
^Δ^FLI	PCSK9i use	−6.8 (−11.5, −2.1)	−0.35	0.006^*^
	SGLT2i use	−1.1 (−4.6, 2.4)	−0.07	0.54
	Baseline FLI	−0.37 (−0.58, −0.16)	−0.42	< 0.001^*^
^Δ^APRI	PCSK9i use	−0.021 (−0.048, 0.006)	−0.18	0.12
^Δ^FIB-4	PCSK9i use	−0.19 (−0.46, 0.08)	−0.16	0.17

PCSK9i, proprotein convertase subtilisin kexin type-9 inhibitor; SGLT-2i, sodium-glucose co-transporter 2 inhibitor; BMI, body mass index; HbA1c, hemoglobin A1c; CAP, controlled attenuation parameter; FLI, fatty liver index; APRI, aspartate aminotransferase to platelet ratio index; FIB-4, fibrosis-4 index. ^Δ^ indicates the reduction from baseline to the 12-week follow-up. ^*^P < 0.05 indicates statistical significance.

## 4 Discussion

This study focuses on the effects of PCSK9i on patients with MAFLD combined with T2DM. Our findings indicate that PCSK9i not only effectively reduce lipid levels but also improve hepatic steatosis surrogate indicators. BMI remained stable with good glycemic control assessed by HbA1c and FBG levels. These benefits may be attributed to the regulation of hepatic lipid metabolism, reduction of insulin resistance, and inhibition of inflammatory and fibrotic signaling pathways, offering new insights and treatment options for managing T2DM with MAFLD and supporting the application of PCSK9i in metabolic liver diseases.

With the improvement of living standards, chronic metabolic diseases have become highly prevalent globally. The incidence of obesity and T2DM is also on the rise (21). Patients with T2DM often experience impaired glucose metabolism due to reduced insulin sensitivity, leading to a cascade of cardiovascular and metabolic diseases. Epidemiological studies have shown that individuals with T2DM are more likely to develop lipid metabolic disorders, consequently increasing the risk of MAFLD (22). Insulin resistance plays a central role in the pathogenesis of both T2DM and MAFLD, with a high prevalence of MAFLD among T2DM patients (5, 23). T2DM with MAFLD not only affects liver function but also complicates glucose management and quality of life. As the disease progresses, insulin resistance and β-cell failure worsen, making glycemic control increasingly challenging (24). Currently, there are no specific treatments for MAFLD, and clinical management of T2DM with MAFLD is often overlooked. Although MAFLD is closely related to hyperlipidemia, the impact of lipid-lowering drugs on MAFLD remains controversial. PCSK9i was initially used to treat familial hypercholesterolemia and later approved for primary and secondary prevention of cardiovascular events in patients who could not achieve target LDL-C levels with optimal statin therapy or were intolerant to statins (25). Recent studies have suggested that PCSK9 played a role in the regulation of T2DM, showing a positively correlation between the elevated PCSK9 levels and the high level of FBG, plasma insulin concentrations, and insulin resistance (IR) (26). Mechanistically, PCSK9 promotes the degradation of the fatty acid transporter CD36, thereby reducing hepatic uptake of free fatty acids (FFAs) and mitigating intrahepatic lipid accumulation (16), indicating that PCSK9i could potentially prevent the progression of MAFLD.

PCSK9i works by binding to PCSK9 in the plasma, preventing its interaction with the low-density lipoprotein receptor (LDL-R) on hepatocytes, thereby reducing the degradation of LDL-R and enhancing the clearance of LDL-C from the bloodstream. This mechanism not only benefits cardiovascular health but also appears to improve MAFLD independently of lowering LDL-C levels (8). In our study, PCSK9i effectively controlled lipid levels in patients with T2DM and MAFLD, significantly reducing TC and LDL-C. The combination of PCSK9i showed better lipid control compared to atorvastatin alone, suggesting that PCSK9i may offer superior management of dyslipidemia. Considering lipid abnormalities is a primary cause of hepatic fat accumulation, PCSK9i group may contribute to reduced hepatic lipid deposition.

Furthermore, PCSK9i may improve insulin resistance and inhibit the NF-κB-mediated inflammatory signaling pathway, thereby synergistically reducing lipotoxicity (27). In animal models of T2DM with fatty liver, PCSK9i significantly inhibited the activation of the TGF-β1/Smad3 pathway and reduced collagen deposition, suggesting that they may directly intervene in fibrosis by regulating the activation of hepatic stellate cells (HSCs) (28, 29). In clinical practice, the use of PCSK9i should be tailored to individual patient needs. For patients with poor lipid control, increasing the dose of PCSK9i or combining them with other lipid-lowering drugs may be considered. For patients with worsening hepatic fat accumulation or fibrosis, additional antifibrotic therapies or referrals to hepatology specialists may be necessary. For patients with MAFLD combined with T2DM, it is essential to closely monitor lipid profiles, blood glucose levels, liver biochemical markers, as well as indicators of hepatic steatosis and fibrosis.

In our study, non-invasive fibrosis and steatosis surrogate indicators (30) such as APRI, FIB-4, APRI and CAP were observed. In Danis et al's (30) study, FibroScan transient elastography demonstrated a statistically significantly superior relationship compared to FIB-4 and APRI. FibroScan transient elastography uses CAP to quantify hepatic fat accumulation accurately (31). Both FLI (32) and CAP (33) reflect the degree of hepatic fat accumulation and have been shown to predict liver steatosis outcomes with varying sensitivity and specificity. In our study, unadjusted analyses revealed that all surrogate indicators APRI, FIB-4, APRI and CAP showed significant reductions in both groups at the 12-week follow-up, with more pronounced improvements in the PCSK9i group. These findings are consistent with those of Scicali et al., (34) who reported significant improvements in fatty liver biomarkers in patients with familial hypercholesterolemia and MAFLD treated with PCSK9i.

Meta-analyses have shown that SGLT-2i (35) and glucagon-like peptide-1 receptor agonists (GLP-1RA) (36) can improve hepatic fat content in patients with T2DM and MAFLD. Our study excluded patients using GLP-1 inhibitors, and there were no significant differences in the use of SGLT-2i between the two groups, suggesting that our results were not influenced by these medications. To address the potential confounding effects of SGLT2i use, we performed additional multivariable regression analyses. After adjustment, PCSK9i use remained independently associated with greater reductions in steatosis surrogate indicators (FLI and CAP), whereas SGLT-2i use was not independently associated with these outcomes. While unadjusted comparisons showed numerical reductions in APRI and FIB-4, the observed reductions in APRI and FIB-4 were attenuated after adjusting for confounders (Table 4), suggesting that PCSK9i's effect on established fibrosis may be limited within a 12-week window. The significant improvements in CAP and FLI primarily reflect reduced steatosis rather than fibrosis regression. In addition, the close relationship between improved glycemic control and reduced hepatic fat accumulation has been demonstrated in multiple studies (35, 36). The reduction in HbA1c observed in both groups at the 12-week follow-up in our study may also have contributed to the observed decreases in hepatic fat content.

This study has several limitations. First, the small sample size and short follow-up period limit the assessment of the long-term efficacy and safety of PCSK9i. Histological endpoints, cardiovascular outcomes and safety beyond 12 weeks remain unknown. Second, baseline creatinine was statistically higher in the PCSK9i group, although both groups had normal renal function. We addressed this by including creatinine as a covariate in multivariable analyses, which did not alter the main conclusions regarding hepatic steatosis surrogate markers. Third, APRI and FIB-4 are surrogate indices that correlate with fibrosis but are also acutely influenced by aminotransferase fluctuations. 12 weeks maybe inadequate to detect true extracellular-matrix remodeling. Longer trials (≥12 months) with serial elastography or liver biopsy are required to verify whether PCSK9-i therapy slows or reverses fibrosis progression. Third, the single-center design may restrict the generalizability of the results. Future studies should increase sample sizes, extend follow-up periods, and employ multicenter randomized controlled designs.

## 5 Conclusions

In summary, PCSK9i demonstrated short-term improvements during the 12-week observation period in lipid profiles and liver steatosis surrogate indicators in patients with T2DM and MAFLD. Their mechanisms of action may involve regulating lipid metabolism, reducing insulin resistance, and inhibition of inflammatory and fibrotic signaling pathways. In clinical practice, the rational use of PCSK9i, combined with individualized patient management and lifestyle interventions, could provide more effective strategies for patients with T2DM and MAFLD, providing a basis for longer-term studies evaluating fibrosis regression. However, further long-term, multicenter randomized controlled studies with larger sample sizes are needed to validate these findings and explore the optimal application of PCSK9i in MAFLD treatment.

## Data Availability

The raw data supporting the conclusions of this article will be made available by the authors, without undue reservation.

## References

[1] Eslam M Sanyal AJ George J. MAFLD: a consensus-driven proposed nomenclature for metabolic associated fatty liver disease. Gastroenterology. (2020) 158:1999–2014.e1. doi: 10.1053/j.gastro.2019.11.312 32044314

[2] Rojas Á Lara-Romero C Muñoz-Hernández R Gato S Ampuero J Romero-Gómez M. Emerging pharmacological treatment options for MAFLD. Ther Adv Endocrinol Metab. (2022) 13:20420188221142452. doi: 10.1177/20420188221142452 36533188 PMC9747889

[3] Habibullah M Jemmieh K Ouda A Haider MZ Malki MI Elzouki AN. Metabolic-associated fatty liver disease: a selective review of pathogenesis, diagnostic approaches, and therapeutic strategies. Front Med. (2024) 11:1291501. doi: 10.3389/fmed.2024.1291501 38323033 PMC10845138

[4] Lim S Kim JW Targher G. Links between metabolic syndrome and metabolic dysfunction-associated fatty liver disease. Trends Endocrinol Metab. (2021) 32:500–14. doi: 10.1016/j.tem.2021.04.008 33975804

[5] Mantovani A Dalbeni A. Treatments for NAFLD: state of art. Int J Mol Sci. (2021) 22:2350. doi: 10.3390/ijms22052350 33652942 PMC7956331

[6] Huang Z Ye D Loomes K Cheng KK Hui HX. Editorial: metabolic associated fatty liver disease: clinical perspectives from pathogenesis to diagnosis and treatment. Front Endocrinol. (2023) 14:1206642. doi: 10.3389/fendo.2023.1206642 37469990 PMC10352314

[7] Jakielska E Głuszak P Walczak M Bryl W. Effects of PCSK9 inhibitors on metabolic-associated fatty liver disease: a short review. Przeglad gastroenterologiczny. (2023) 18:148–53. doi: 10.5114/pg.2023.126054 37538291 PMC10395066

[8] Theocharidou E Papademetriou M Reklou A Sachinidis A Boutari C Giouleme O. The role of PCSK9 in the pathogenesis of non-alcoholic fatty liver disease and the effect of PCSK9 inhibitors. Curr Pharm Des. (2018) 24:3654–7. doi: 10.2174/1381612824666181010123127 30317984

[9] Meade R Ibrahim D Engel C Belaygorod L Arif B Hsu FF . Targeting fatty acid synthase reduces aortic atherosclerosis and inflammation. Commun Biol. (2025) 8:262. doi: 10.1038/s42003-025-07656-1 39972116 PMC11840040

[10] Lambert G Charlton F Rye KA Piper DE. Molecular basis of PCSK9 function. Atherosclerosis. (2009) 203:1–7. doi: 10.1016/j.atherosclerosis.2008.06.010 18649882

[11] Demers A Samami S Lauzier B Des Rosiers C Ngo Sock ET Ong H . PCSK9 Induces CD36 degradation and affects long-chain fatty acid uptake and triglyceride metabolism in adipocytes and in mouse liver. Arterioscler Thromb Vasc Biol. (2015) 35:2517–25. doi: 10.1161/ATVBAHA.115.306032 26494228

[12] Fassi EMA Citarella A Albani M Milano EG Legnani L Lammi C . PCSK9 inhibitors: a patent review 2018-2023. Expert Opin Ther Pat. (2024) 34:245–61. doi: 10.1080/13543776.2024.2340569 38588538

[13] Wang X Wen D Chen Y Ma L You C. PCSK9 inhibitors for secondary prevention in patients with cardiovascular diseases: a Bayesian network meta-analysis. Cardiovasc Diabetol. (2022) 21:107. doi: 10.1186/s12933-022-01542-4 35706032 PMC9202167

[14] Shakir A Barron K Modi K. Qualitative and quantitative effects of PCSK9 inhibitors in familial hypercholesterolemia: a synthetic review. Curr Probl Cardiol. (2023) 48:101550. doi: 10.1016/j.cpcardiol.2022.101550 36529229

[15] Baragetti A Balzarotti G Grigore L Pellegatta F Guerrini U Pisano G . PCSK9 deficiency results in increased ectopic fat accumulation in experimental models and in humans. Eur J Prev Cardiol. (2017) 24:1870–7. doi: 10.1177/2047487317724342 28758421

[16] Lebeau PR Byun JH Platko K Al-Hashimi AA Lhoták Š MacDonald ME . Pcsk9 knockout exacerbates diet-induced non-alcoholic steatohepatitis, fibrosis and liver injury in mice. JHEP Rep. (2019) 1:418–29. doi: 10.1016/j.jhepr.2019.10.009 32039393 PMC7005770

[17] Bjørklund MM Bernal JA Bentzon JF. Atherosclerosis induced by adeno-associated virus encoding gain-of-function PCSK9. Methods Mol Biol. (2022) 2419:461–73. doi: 10.1007/978-1-0716-1924-7_27 35237981

[18] Shafiq M Walmann T Nutalapati V Gibson C Zafar Y. Effects of proprotein convertase subtilisin/kexin type-9 inhibitors on fatty liver. World J Hepatol. (2020) 12:1258–66. doi: 10.4254/wjh.v12.i12.1258 33442452 PMC7772734

[19] Rinella ME Neuschwander-Tetri BA Siddiqui MS Abdelmalek MF Caldwell S Barb D . AASLD practice guidance on the clinical assessment and management of nonalcoholic fatty liver disease. Hepatology. (2023) 77:1797–835. doi: 10.1097/HEP.0000000000000323 36727674 PMC10735173

[20] ElSayed NA Aleppo G Aroda VR Bannuru RR Brown FM Bruemmer D . 3. prevention or delay of type 2 diabetes and associated comorbidities: standards of care in diabetes-2023. Diabetes care. (2023) 46:S41–8. doi: 10.2337/dc23-S003 36507633 PMC9810464

[21] Ruze R Liu T Zou X Song J Chen Y Xu R . Obesity and type 2 diabetes mellitus: connections in epidemiology, pathogenesis, and treatments. Front Endocrinol. (2023) 14:1161521. doi: 10.3389/fendo.2023.1161521 37152942 PMC10161731

[22] Xie J Huang H Liu Z Li Y Yu C Xu L . The associations between modifiable risk factors and nonalcoholic fatty liver disease: a comprehensive Mendelian randomization study. Hepatology. (2023) 77:949–64. doi: 10.1002/hep.32728 35971878

[23] Stefan N Cusi K. A global view of the interplay between non-alcoholic fatty liver disease and diabetes. Lancet Diabetes Endocrinol. (2022) 10:284–96. doi: 10.1016/S2213-8587(22)00003-1 35183303

[24] Lomonaco R Bril F Portillo-Sanchez P Ortiz-Lopez C Orsak B Biernacki D . Metabolic impact of nonalcoholic steatohepatitis in obese patients with type 2 diabetes. Diabetes Care. (2016) 39:632–8. doi: 10.2337/dc15-1876 26861926 PMC5864108

[25] Schmidt AG Carter JL Pearce LS Wilkins JT Overington JP Hingorani AD . PCSK9 monoclonal antibodies for the primary and secondary prevention of cardiovascular disease. Cochrane Database Syst Rev. (2020) 10:Cd011748. doi: 10.1002/14651858.CD011748.pub3 33078867 PMC8094613

[26] Macchi C Ferri N Sirtori CR Corsini A Banach M Ruscica M. Proprotein convertase subtilisin/kexin type 9: a view beyond the canonical cholesterol-lowering impact. Am J Pathol. (2021) 191:1385–97. doi: 10.1016/j.ajpath.2021.04.016 34019847

[27] Tang ZH Peng J Ren Z Yang J Li TT Li TH . New role of PCSK9 in atherosclerotic inflammation promotion involving the TLR4/NF-κB pathway. Atherosclerosis. (2017) 262:113–22. doi: 10.1016/j.atherosclerosis.2017.04.023 28535426

[28] He H Zhong Y Wang H Tang PM Xue VW Chen X . Smad3 mediates diabetic dyslipidemia and fatty liver in db/db mice by targeting PPARδ. Int J Mol Sci. (2023) 24:1396. doi: 10.3390/ijms241411396 37511155 PMC10380492

[29] Ning L Zou Y Li S Cao Y Xu B Zhang S . Anti-PCSK9 treatment attenuates liver fibrosis via inhibiting hypoxia-induced autophagy in hepatocytes. Inflammation. (2023) 46:2102–19. doi: 10.1007/s10753-023-01865-8 37466835 PMC10673768

[30] Danis N Gunsar F Yilmaz F Nart D Turan I Karasu Z . Performance of non-invasive fibrosis markers in biopsy-proven liver disorders. Hepatol Forum. (2025) 6:16–21. doi: 10.14744/hf.2024.2024.0024 40255951 PMC12008458

[31] Desai GS Hajare S Ghorpade S. Incidence of non-alcoholic fatty liver disease (NAFLD)/non-alcoholic steatohepatitis (NASH) in the female population of North Karnataka: a cross-sectional study. Cureus. (2024) 16:e66257. doi: 10.7759/cureus.66257 39238753 PMC11375481

[32] Crudele L De Matteis C Novielli F Di Buduo E Petruzzelli S De Giorgi A . Fatty liver index (FLI) is the best score to predict MASLD with 50% lower cut-off value in women than in men. Biol Sex Differ. (2024) 15:43. doi: 10.1186/s13293-024-00617-z 38760802 PMC11100212

[33] Kabisch S Bäther S Dambeck U Kemper M Gerbracht C Honsek C . Liver fat scores moderately reflect interventional changes in liver fat content by a low-fat diet but not by a low-carb diet. Nutrients. (2018) 10:157. doi: 10.3390/nu10020157 29385034 PMC5852733

[34] Scicali R Di Pino A Urbano F Ferrara V Marchisello S Di Mauro S . Analysis of steatosis biomarkers and inflammatory profile after adding on PCSK9 inhibitor treatment in familial hypercholesterolemia subjects with nonalcoholic fatty liver disease: a single lipid center real-world experience. Nutr Metab Cardiovasc Dis. (2021) 31:869–79. doi: 10.1016/j.numecd.2020.11.009 33549441

[35] Wong C Yaow CYL Ng CH Chin YH Low YF Lim AYL . Sodium-glucose co-transporter 2 inhibitors for non-alcoholic fatty liver disease in Asian patients with type 2 diabetes: a meta-analysis. Front Endocrinol. (2020) 11:609135. doi: 10.3389/fendo.2020.609135 33643221 PMC7905212

[36] Wong C Lee MH Yaow CYL Chin YH Goh XL Ng CH . Glucagon-like peptide-1 receptor agonists for non-alcoholic fatty liver disease in type 2 diabetes: a meta-analysis. Front Endocrinol. (2021) 12:609110. doi: 10.3389/fendo.2021.609110 33897616 PMC8063104

